# Effects of anterior approach to quadratus lumborum block on postoperative cognitive function following hip surgery in older people: a randomized controlled clinical trial

**DOI:** 10.1186/s12877-023-04514-9

**Published:** 2023-12-11

**Authors:** Manhua Zhu, Yuliu Mei, Ruifen Zhou, Lingzhi Wang, Xiaoyan Zhang

**Affiliations:** 1 Department of Anesthesiology, Ningbo Medical Center Lihuili Hospital, Ningbo University, No. 1111 jiangnan Road, Ningbo, 315040 Zhejiang China; 2 Department of Anesthesiology, Ningbo Beilun People’s Hospital, No 1288 lushan east Road, Ningbo, 315800 Zhejiang China

**Keywords:** Quadratus Lumborum block, Postoperative cognitive dysfunction, Hip Surgery, Older people

## Abstract

**Background:**

Peripheral nerve block, including the quadratus lumborum block (QLB), has been used for postoperative analgesia in hip surgery. However, the effects of QLB on cognitive function after hip surgery remain unknown. This study aimed to assess the effects of the anterior approach to QLB on postoperative cognitive function in older people undergoing hip surgery.

**Methods:**

Sixty older people who underwent hip surgery from May 2021 to May 2022 were randomly divided into the QLB (n = 30) and control groups (n = 30). The Montreal Cognitive Assessment (MoCA) score (mean ± SD) was measured one day preoperatively and seven and 30 days postoperatively. The frequency (%) of postoperative cognitive dysfunction (POCD) was examined seven and 30 days postoperatively. The visual analog scale (VAS) scores at rest and Bruggrmann comfort scale (BCS) scores [Median (IQR)] 6 h (t1), 12 h (t2), 24 h (t3), and 48 h (t4) after surgery were assessed. The plasma high mobility group box protein 1 (HMGB1) and levels of interleukin-6 (IL-6) (mean ± SD) were evaluated 1 h preoperatively (baseline) and 24 h postoperatively (day 1). The requirement for rescue analgesia [Median (IQR)], time to first off-bed activity (mean ± SD), and adverse effects after surgery were also recorded.

**Results:**

Compared with the control group, the frequency of POCD was significantly lower in the QLB group seven days postoperatively (10.7% vs. 34.5%, *P* = 0.033), but no difference at 30 days postoperatively (3.6% vs. 10.3%, *P* = 0.319). There was no significant difference in MoCA scores between the two groups at one day preoperatively and 30 days postoperatively. However, the MoCA scores at seven days postoperatively were higher in the QLB group than in the control group (27.4 ± 1.81 vs. 26.4 ± 1.83, *P* = 0.043). In the QLB group, the VAS scores at t1, t2, and t3 were lower [3(2–4) vs. 4(3–4), *P* = 0.028; 3(2–3) vs. 4(3–5), *P* = 0.009; 2(1–3) vs. 2(2–3), *P* = 0.025], and the BCS scores at t1, t2, and t3 were higher than those in the control group [3(1–3) vs. 1(1–2), *P* = 0.006; 3(2–3) vs. 2(1–3), *P* = 0.011; 3(2–4) vs. 2(2–3), *P* = 0.041]. The patients in the QLB group reported significantly fewer requirements for rescue analgesia [0(0–1) vs. 1(0–2), *P* = 0.014]. The plasma levels of HMGB1 and IL-6 at 24 h postoperatively in the QLB group were significantly lower than in the control group (749.0 ± 185.7 vs. 842.1 ± 157.9, *P* = 0.046; 24.8 ± 8.1 vs. 31.9 ± 5.5, *P* < 0.001). The time to first off-bed activity from the end of surgery was shorter in the QLB group (25.3 ± 5.3 vs. 29.7 ± 6.9, *P* = 0.009). There was no significant difference in the incidence of postoperative complications between the two groups.

**Conclusions:**

Anterior QLB given to older people undergoing hip surgery could promote early postoperative cognitive function recovery, provide adequate postoperative analgesia, and inhibit the release of inflammatory factors.

**Trial registration:**

Chictr.org.cn identifier ChiCTR2000040724 (Date of registry: 08/12/2020, prospectively registered).

## Background

Hip fractures are more common in older people due to osteoporosis. It is estimated that around six million patients worldwide will suffer hip fractures annually by 2050 as the population ages [1]. Clinically, hip surgery is a common and effective treatment for hip fractures. There will be an increasing number of older people undergoing hip surgery, including osteosynthesis and arthroplasty. However, severe surgical trauma, postoperative pain, and postoperative cognitive dysfunction (POCD) can be a considerable challenge for older people undergoing hip surgery [2, 3]. POCD is a common complication following hip surgery, with a median frequency of 19.3% at one week and 10% at three months [4]. It refers to a series of neurological complications, such as attention deficit and intellectual and memory impairment after surgery, which seriously affect a patient’s postoperative recovery and quality of life [5]. Age, surgical stress, postoperative pain, anesthesia mode, and postoperative inflammatory reaction are risk factors for POCD [6]. It has been reported that effective pre-emptive pain management by femoral nerve block can reduce postoperative pain and improve patient cognitive function in patients undergoing total knee arthroplasty [7]. Surgery can activate a patient’s immune system and produce a peripheral inflammatory response. Increased release of inflammatory cytokines, such as interleukin-6 (IL-6), tumor necrosis factor-α (TNF-α), and high mobility group box protein 1 (HMGB1), after surgery can impair cognitive function, resulting in POCD [8].

Based on the expert consensus of the Chinese Association of Anesthesiologists, multimodal analgesia is recommended to alleviate postoperative pain, reduce complications, and improve postoperative recovery quality. As a trunk nerve block, quadratus lumborum block (QLB) has been widely used for postoperative analgesia in patients undergoing abdominal and lower limb surgeries [9]. QLB can provide adequate analgesia and reduce opioid requirements after total hip arthroplasty [10]. Sufficient postoperative pain control is essential for reducing operative stress, inflammation, and POCD, which is beneficial to postoperative rehabilitation [11]. To the best of our knowledge, no study has evaluated the effects of QLB on postoperative cognitive function following hip surgery. This study aimed to investigate the effects of the anterior approach to QLB on postoperative cognitive function, postoperative analgesia, and inflammatory cytokines in older people undergoing hip surgery.

## Methods

**Fig. 1:**
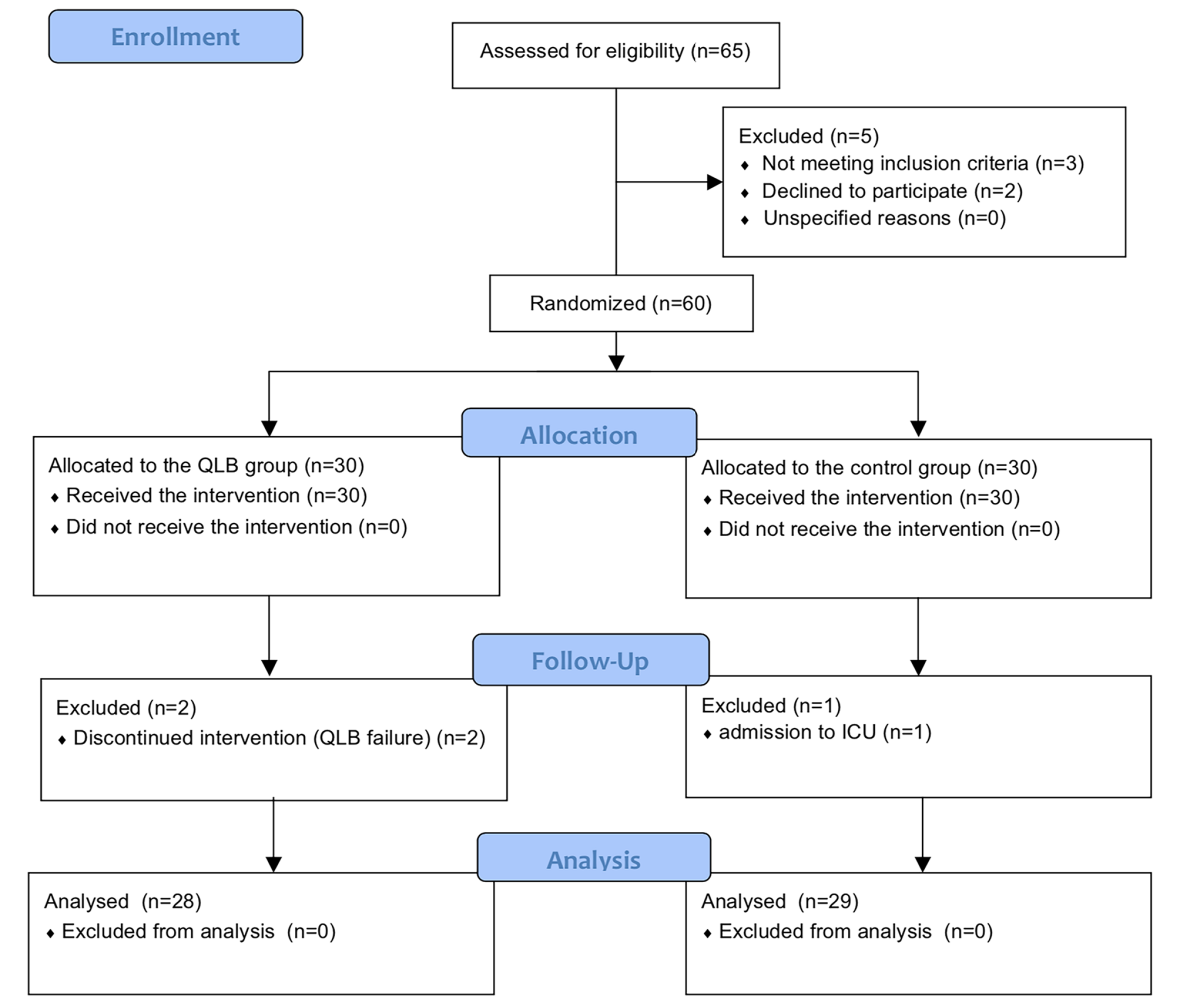
Flow chart of the participants

This randomized controlled study was carried out in a single institution and was approved by the Medical Ethics Committee of our hospital (KY2021PJ067). It was registered at www.chictr.org.cn (ChiCTR2000040724) before the participant’s enrollment. Sixty patients undergoing hip surgery from May 2021 to May 2022 in Ningbo Medical Center Lihuili Hospital were enrolled after obtaining written informed consent. All subjects were randomly divided into the QLB and control groups using a random number table method (Fig. 1). Inclusion criteria: age > 65 years; American Society of Anesthesiologists (ASA) I-II; received primary school education or above; Montreal Cognitive Assessment (MoCA) score ≥ 26 before surgery; scheduled for hip surgery. Exclusion criteria: contraindications to neuraxial anesthesia; history of mental disease; severe circulatory, respiratory or nervous system diseases; long-term use of antidepressants or narcotic analgesics; alcohol abuse; inability to communicate appropriately; and hypersensitivity to local anesthetics.

**Fig. 2:**
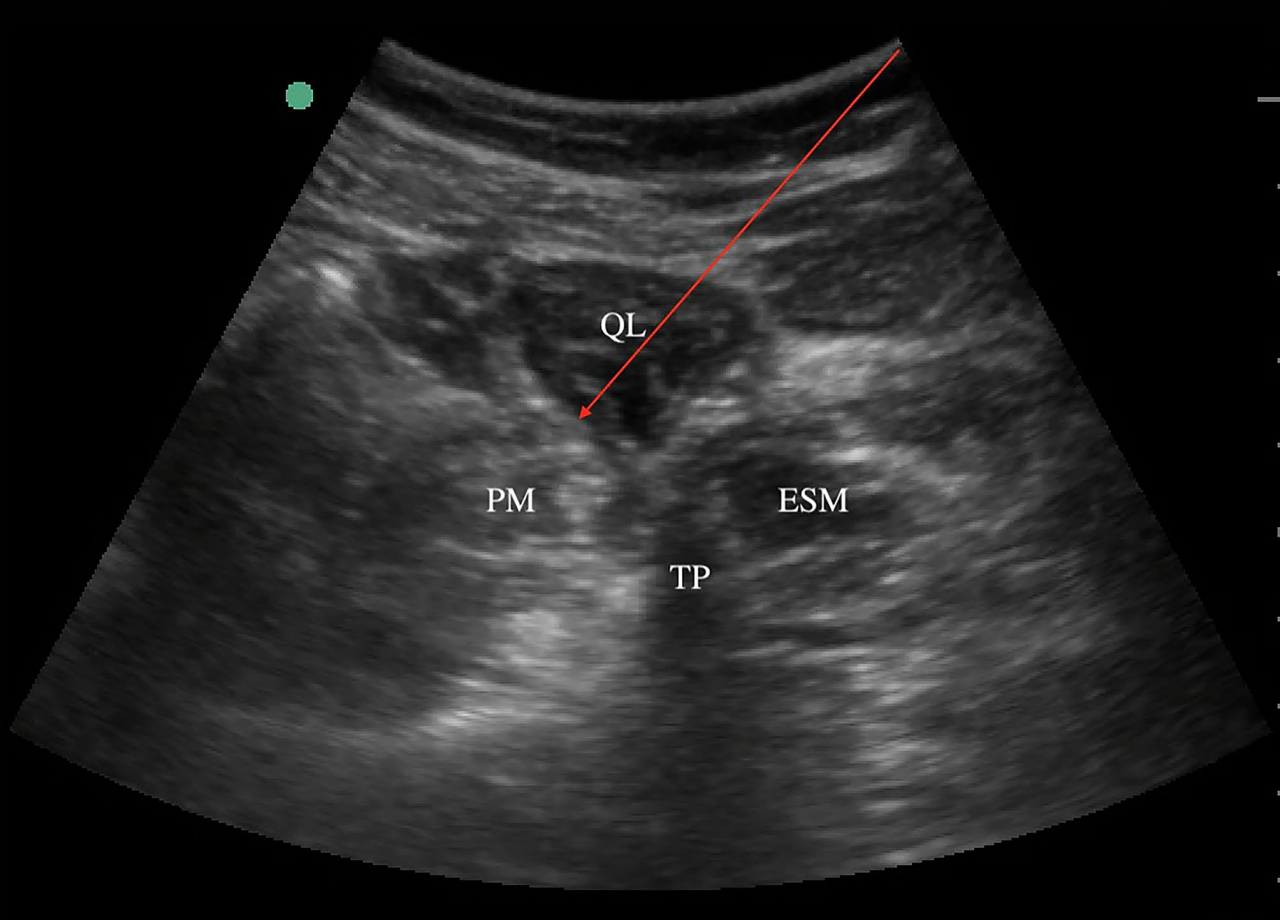
Ultrasound image of anterior QLB. The arrow indicates the trajectory of the needle. QL, quadratus lumborum; PM, psoas major; ESM, erector spinae muscles; TP, transverse process

Standard monitoring consisted of a 5-lead electrocardiogram (ECG), pulse oxygen saturation (SpO_2_), and invasive radial arterial blood pressure applied in the operating room. An intravenous line was established. All patients were ultrasonically scanned in a lateral decubitus position with the surgical side up before anesthesia. A curvilinear probe (2–5 HZ, Edge, Sonosite, Seattle, USA) was positioned above the iliac crest at the level of L3 and adjusted until the L3 transverse process, quadratus lumborum (QL) muscle, erector spinae, and psoas major were identified and presented “Shamrock Sign.” Then patients in the QLB group received an ultrasound-guided anterior approach to QLB. Following disinfection and the puncture, the point was infiltrated with 1% lidocaine (1 mL), a 21 gauge×100 mm SonoPlex Stim needle (Pajunk, Geisingen, Germany) was penetrated in the posterolateral to the anteromedial direction and advanced to the anterior part of the QL muscle, 30 mL of 0.3% ropivacaine (Naropin, AstraZeneca AB Company, Södertälje, Sweden) was injected slowly between QL muscle and psoas major. Local anesthetic (LA) spread was observed (Fig. 2). In contrast, the patients in the control group only received ultrasound scanning to identify QL muscle and subcutaneous injection with 1% lidocaine (1 mL) but without nerve block needle insertion. A senior anesthesiologist performed all blocks.

After that, all patients were subjected to spinal anesthesia in the lateral position. An epidural needle was inserted at L2–L3 or L3–L4 intervertebral space. After reaching the epidural space, a spinal needle was inserted into the subarachnoid space through the epidural needle. A 0.75% ropivacaine (Naropin, AstraZeneca AB Company, Södertälje, Sweden) (1.1–1.8 mL) was administered to achieve a sensory block to the level of T10 dermatome. Then, a catheter was placed in the epidural space. Routine perioperative care was provided to all patients in the operating room. Parecoxib sodium (40 mg) was administered intravenously 15 min before the end of the surgery for postoperative analgesia. Intravenous patient-controlled intravenous analgesia pump with 7 mg/mL tramadol and 0.1 mg/mL tropisetron was administered to all patients. Other routine postoperative pain management consisted of parecoxib sodium (40 mg) twice daily. An intravenous dezocine (5 mg) was used for rescue analgesia if the VAS scores ≥ 4.

### Measurements

The primary outcome measure was the frequency (in %) of the occurrence of POCD, defined according to changes in the MoCA scores. The secondary outcome measures were VAS scores at rest and BCS scores 6 h (t1), 12 h (t2), 24 h (t3), and 48 h (t4) after surgery; the plasma levels of HMGB1 and IL-6; the requirement for rescue analgesia; the time to first off-bed activity; and adverse effects after surgery (postoperative nausea and vomiting [PONV], urinary retention, wound swelling, deep vein thrombosis, hematoma, and LA systemic toxicity [LAST]).

All patients received the neuropsychological test in a quiet environment by a well-trained anesthesiologist using the MoCA one day preoperatively, seven and 30 days postoperatively. The MoCA [12] is a rapid cognitive screening tool with high sensitivity. It is widely used in clinical practice, including visuospatial ability, naming, short-term memory, attention, language, abstraction, delayed recall, and orientation. The MoCA Beijing version was adopted in the study. As mentioned in previous studies, a postoperative MoCA score ≥ 1 standard deviation (SD) lower than the preoperative score indicated POCD [13].

The pain scores on the visual analog scale (VAS) (0, no pain, 10 excruciating pain) at rest, and comfort scores on the Bruggrmann comfort scale (BCS) (0, continuous pain; 1, painless at rest, severe pain while deep breathing or coughing; 2, painless at rest, slight pain while deep breathing or coughing; 3, painless when deep breathing; 4, painless when coughing) was measured 6 h (t1), 12 h (t2), 24 h (t3), and 48 h (t4) after surgery. The requirement for rescue analgesia was also recorded 48 h after surgery.

Venous blood samples (5 mL) were collected from all patients 1 h preoperatively (baseline) and 24 h postoperatively (day 1). The samples were put into sterile heparin tubes immediately and centrifuged at 1000×g for 15 min at 4 °C, and the plasma was extracted and stored at − 80 °C for future assays. The plasma concentrations of HMGB1 and IL-6 were tested by a professional biotechnology company (Animalunion Biotechnology Co., Ltd., Shanghai, China) using the ELISA method.

In addition, the time to first off-bed activity from the end of surgery, and patients’ self-reported adverse effects (PONV, urinary retention, wound swelling, deep vein thrombosis, hematoma, and LAST) were also recorded 24 h after surgery. All clinical data were collected by staff unaware of the patient’s grouping.

### Statistical analyses

The power analysis for this study was based on a pilot study, which showed a POCD (seven days postoperatively) rate of 5% in the QLB group and 35% in the control group. The pilot study was a randomized controlled study with 20 patients in each group. A sample size calculation using IBM SPSS Sample Power version 3.0 (IBM Corp., Armonk, New York, USA) showed that a sample of 24 participants was required in each group to achieve a statistical power of 0.8 with a significance level alpha of 0.05. Considering the 20% drop-out rate, we increased the number of participants to 30 in each group.

SPSS V.24.0 (IBM Corp., Armonk, New York, USA) was used to conduct statistical analyses. Normality was assessed using the Kolmogorov-Smirnov test. Normally distributed data were expressed as the mean and SD, non-normally distributed data were expressed as median and quartiles, and categorical data were expressed as frequency and percentages. Intragroup comparisons were analyzed using the Student’s t-test, while differences in non-normally distributed data were assessed using the Mann–Whitney U test. Categorical data were analyzed using the chi-square (*χ2*) test or Fisher’s exact test. A repeated-measures analysis of variance was applied for comparisons between the two groups at different time points. A *P*-value of < 0.05 was considered statistically significant.

## Results

After screening, a total of 65 patients were recruited for the study. Three patients were excluded for not meeting the inclusion criteria, and two declined to participate. Sixty patients were randomly assigned to the QLB or control groups. Two patients in the QLB group were excluded for failed quadratus lumborum block, one was excluded in the control group for unexpectedly admitting to ICU. None of the three patients were included in the analysis. The final analysis included 28 patients in the QLB group and 29 in the control group. Patient demographic data (age, sex, body mass index, and education) and clinical characteristics (ASA status, comorbidities, type of surgery, and operative time) were comparable between the two groups (Table 1).

**Table 1:** Demographics and clinical characteristics of the patients

		QLB group (n = 28)	Control group (n = 29)
Age (years)		74.8 ± 6.2	75.6 ± 7.6
Sex ratio [n (%)]	male	16 (57.1%)	18 (62.1%)
BMI (kg/m^2^)		22.2 ± 3.1	20.8 ± 3.2
Education (years)		7.4 ± 2.4	7.0 ± 2.0
ASA [n (%)]			
I II		10 (35.7%) 18 (64.3%)	8 (27.6%) 21 (72.4%)
Comorbidities [n (%)]			
Hypertension Diabetes		13 (46.4%) 8 (27.6%)	15 (51.7%) 7 (24.1%)
Type of surgery [n (%)]			
Osteosynthesis Arthroplasty		10 (35.7%) 18 (64.4%)	13 (44.8%) 16 (55.2%)
Operative time (min)		73.4 ± 20.6	79.8 ± 15.6

The data are represented as the mean ± SD or number (%)

BMI = Body mass index; ASA = American Society of Anesthesiologists

### Primary outcome

**Fig. 3:**
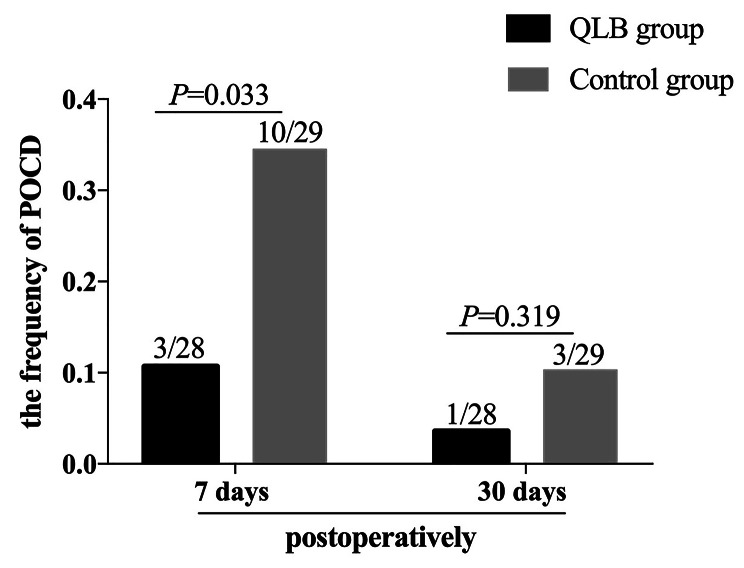
Postoperative cognitive dysfunction (POCD) at seven and 30 days postoperatively between the two groups. Data were compared by Fisher’s exact test

POCD occurred in three patients (10.7%) in the QLB group vs. in ten patients (34.5%) in the control group at seven days postoperatively (*P* = 0.033, Odds ratio:0.228). There was no significant difference in the frequency of POCD between the two groups at 30 days postoperatively (QLB group: 3.6% vs. the control group: 10.3%, *P* = 0.319, Odds ratio:0.321; Fig. 3).

A repeated-measures ANOVA was applied for comparisons MoCA between the two groups. The intergroup effect, time effect, and interaction effect had statistically significant (*P* < 0.05). Through simple effect analysis, there was no significant difference in MoCA scores between the two groups one day preoperatively and 30 days postoperatively. However, patients in the QLB group had higher MoCA scores than patients in the control group seven days postoperatively (*P* = 0.043; Table 2).

**Table 2:** The Montreal Cognitive Assessment (MoCA) scores of the patients

	Group	n	1 day preoperatively	7 days postoperatively	30 days postoperatively	*P*	${\eta }_{p}^{2}$
MoCA	QLB	28	28.4 ± 2.01	27.4 ± 1.81^#^	27.6 ± 1.57		
	Control	29	28.1 ± 1.92	26.4 ± 1.83	27.3 ± 1.70		
intergroup effect						0.034	0.08
time effect						< 0.001	0.47
interaction effect						0.047	0.06

The data are represented as the mean ± SD

MoCA = Montreal Cognitive Assessment

Compared with the control group, ^#^*P* < 0.05. Data were compared by repeated-measures analysis of variance. ${\eta }_{p}^{2}=\frac{{SS}_{treatment}}{{SS}_{total}+{SS}_{error}}$

### Secondary outcomes

As shown in Table 3, the VAS scores at rest at t1, t2, and t3 were statistically significantly lower in the QLB group than in the control group (*P* < 0.05). The BCS scores were statistically significantly higher in the QLB group compared to the control group at t1, t2, and t3 (*P* < 0.05). However, the VAS and BCS scores did not differ significantly between the two groups at t4 (*P* > 0.05). The patients in the QLB group reported significantly fewer requirements for rescue analgesia than the control group 48 h after surgery (*P* < 0.05, Table 3).

**Table 3:** The postoperative pain scores at rest, comfort scores, and rescue analgesia requirements of the patients [score, M(IQR)].

	QLB group (n = 28)	Control group (n = 29)	*P*
VAS at rest (0–10)			
t1	3(2–4) ^#^	4(3–4)	0.028
t2	3(2–3) ^#^	4(3–5)	0.009
t3	2(1–3) ^#^	2(2–3)	0.025
t4	2(1–2)	2(1–2)	0.664
BCS (0–4)			
t1	3 (1–3) ^#^	1 (1–2)	0.006
t2	3 (2–3) ^#^	2 (1–3)	0.011
t3	3 (2–4) ^#^	2 (2–3)	0.041
t4	3 (3–4)	3 (3–4)	0.763
Rescue analgesia, time			
0–48 h after surgery	0 (0–1) ^#^	1 (0–2)	0.014

The data are represented as the median (interquartile range)

VAS = visual analog scale; BCS = Bruggemann comfort scale

Compared with the control group, ^#^*P* < 0.05. Data were compared by Mann-Whitney U test

**Fig. 4:**
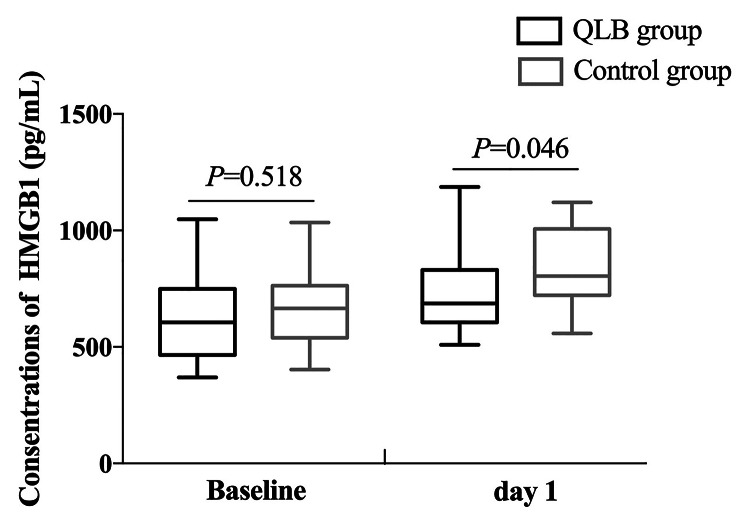
The plasma concentrations of HMGB1 at baseline and day one between the two groups. Data were compared by repeated-measures analysis of variance

**Fig. 5:**
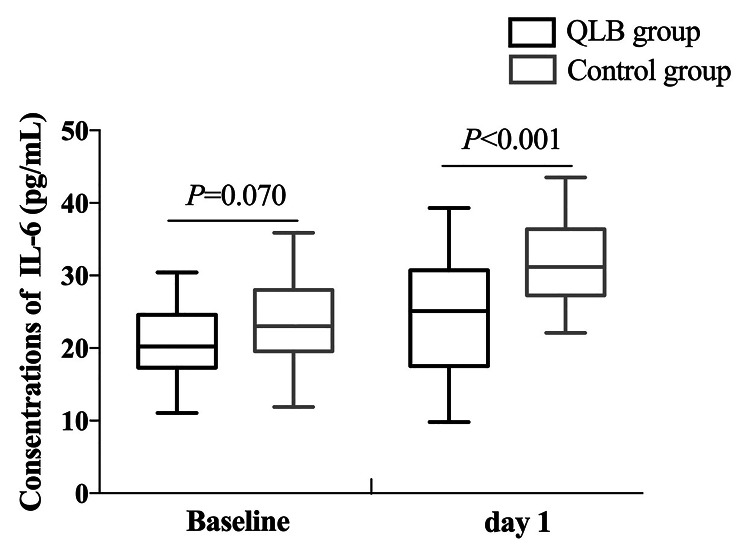
The plasma concentrations of IL-6 at baseline and day one between the two groups. Data were compared by repeated-measures analysis of variance

The plasma concentrations of HMGB1 and IL-6 were comparable between the two groups at baseline (*P* > 0.05), and they presented similar trends in both groups, increasing at day one compared to those at baseline. Compared with the control group, the concentrations of HMGB1 and IL-6 in the QLB group were dramatically lower at day one (*P* < 0.05; Table 4; Figs. 4 and 5).

**Table 4:** The plasma concentrations (pg/mL) of HMGB1 and IL-6 at different time points

	time point	QLB group (n = 28)	Control group (n = 29)	*P*	Cohen’s d
HMGB1	Baseline day 1	638.3 ± 183.5 749.0 ± 185.7^#^	667.9 ± 159.4 842.1 ± 157.9	0.518 0.046	0.172 0.540
IL-6	Baseline day 1	20.8 ± 5.1 24.8 ± 8.1^#^	23.4 ± 5.5 31.9 ± 5.5	0.070 < 0.001	0.490 1.026

The data are represented as the mean ± SD

Compared with the control group, ^#^*P* < 0.05. Data were compared by repeated-measures analysis of variance

Two patients in the QLB group were excluded due to failed QLB block, one of which was overweight and quadratus lumborum (QL) muscle, erector spinae, and psoas major couldn’t be identified well, and the other was due to a large rash at the puncture site. The patients in the QLB group had a shorter time to first off-bed activities from the end of surgery than in the control group (*P* < 0.05). No significant differences were observed in the incidence of adverse effects 24 h after surgery, including PONV, urinary retention, wound swelling, deep vein thrombosis, hematoma, and LAST between them (*P* > 0.05, Table 5).

**Table 5:** Time to first off-bed and postoperative complications of the patients

	QLB group (n = 28)	Control group (n = 29)	*P*	Cohen’s d/Odds ratio
Time to first off-bed from the end of surgery (h)	25.3 ± 5.3^#^	29.7 ± 6.9	0.009	0.715
PONV	3 (10.7%)	4 (13.8%)	0.723	0.750
Urinary retention	2 (7.1%)	3 (10.3%)	0.669	0.641
Wound swelling	2 (7.1%)	1 (3.4%)	0.532	2.148
Deep vein thrombosis	0	0		
Hematoma	0	0		
LAST	0	0		

The data are represented as the mean ± SD or number (%)

PONV = postoperative nausea and vomiting; LAST = local anesthetic systemic toxicity

Compared with group C, ^#^*P* < 0.05. Continuous data were compared by Student’s t-test; categorical data were compared by Fisher’s exact test

## Discussion

As common complications in the hip fracture older people population, neurological complications seriously threaten postoperative rehabilitation, decrease mobility, and increase the length of hospital stay [14]. POCD mainly manifests as reduced concentration, personality changes, and impaired memory. The frequency of POCD in patients with hip fractures was reported with a wide range of 6.7–75% one week after surgery and 8–45% three months after surgery [4]. The high frequency of POCD is attributed to the increasing number of hip surgeries performed on older people. In the current study, the frequency of POCD at seven days postoperatively in the QLB and control group was 10.7% and 34.5%, respectively. The POCD rate in the control group in our study is similar to the reported rate in aged patients following hip surgery (27.3%) [15] but much higher than the results reported by Konishi et al. (15%). [16]. Such a phenomenon may be caused by different populations, the definition of POCD, and surgery type or anesthesia method.

The MoCA is a commonly used cognitive screening method for patients in the early stages of neurocognitive disorders [17]. The results of our study demonstrated that the MoCA scores in the QLB group increased at seven days postoperatively compared with the control group. However, no difference was observed at 30 days postoperatively. The above results suggested that QLB may improve early postoperative cognitive function in older people undergoing hip surgery but had no significant effect on long-term cognitive function.

It has been proved that both preoperative chronic and postoperative acute pain can impair postoperative cognitive function [18]. Providing patients with adequate analgesia can promote early postoperative rehabilitation after hip surgery [19]. The results of the present study showed superior early postoperative analgesia and patient comfort with QLB for hip surgery resulting in lower VAS scores, higher BSC scores, and a reduced number of rescue analgesia compared with the control group. Our findings were in accordance with the study by Kukreja et al. [10], who reported that anterior QLB could provide adequate analgesia after total hip arthroplasty. Better pain control can minimize complications after hip surgery ranging from impairment of cognitive to functional loss [20], which may be one reason for the better early postoperative cognition in the QLB group compared with the control group.

It has been widely recognized that inflammatory responses play an essential role in the pathogenesis of POCD [21]. Cytokines, such as HMGB1 and IL-6, are pivotal in triggering neuroinflammation after surgery in rodent models [22, 23]. Previous research showed that patients with POCD after cytoreductive surgery had higher serum levels of HMGB1 than those without POCD [24]. As an essential pro-inflammatory cytokine, enhanced IL-6 levels are associated with surgical trauma and can cause cognitive deficits [25]. In the present study, the plasma HMGB1 and IL-6 concentrations in patients of the QLB group one day after surgery were significantly lower than those in the control group, suggesting that anterior QLB for hip surgery may interrupt postoperative systemic inflammatory response and subsequently improve cognitive function.

Compared to the control group, the patients of the QLB group had a shorter time to first off-bed activity. This indicated that adequate postoperative analgesia provided by QLB could promote early postoperative rehabilitation of patients, consistent with a previous study performed by Kelly et al. [26]. Hematoma and LAST are severe QLB-related complications. No severe complications were observed in this study, possibly because the direction of the needle and LA diffusion can be seen accurately by ultrasound guidance, thus reducing the incidence of complications. However, the safety of the anterior approach to QLB still needs to be further confirmed by large sample studies.

Also, there were certain limitations to this study. First, sensory testing in the QLB group was not performed to avoid unblinding. Second, the sample size was limited, and only MoCA testing was adopted to assess POCD in this study. Therefore, larger sample sizes and multiple tests with different domains of cognition should be performed in further research to gather more evidence. Third, intention-to-treat analysis should be adopted to evaluate the therapeutic effect or exclusion criteria should be modified to ensure that all subjects are eligible for QLB to improve the comparability of experimental results. Last, a dose of 90 mg of ropivacaine was used in this study, and no systemic toxicity was observed. Nevertheless, the ideal LA dosing, volumes, and optimal injection site of QLB in older people still need to be verified.

## Conclusions

Anterior QLB could accelerate the recovery of early postoperative cognitive function in older people undergoing hip surgery, which may be associated with adequate postoperative analgesia and reduced plasma levels of HMGB1 and IL-6.

## Data Availability

The datasets used during the current study are available from the corresponding author on reasonable request.

## Abbreviations

ASA: America Society of Anesthesiologist
BCS: Bruggrmann comfort scale
BMI: Body mass index
ECG: Electrocardiogram
ELISA: Enzyme-linked immunosorbent assay
HMGB1: High mobility group box protein 1
ICU: Intensive care unit
LA: Local anesthetic
IL-6: Interleukin-6
LAST: Local anesthetic systemic toxicity
MoCA: Montreal Cognitive Assessment
POCD: Postoperative cognitive dysfunction
PONV: Postoperative nausea and vomiting
QL: Quadratus lumborum
QLB: Quadratus lumborum block
SD: Standard deviation
SpO_2_: Percutaneous oxygen saturation
VAS: Visual analogue scale
TNF-α: Tumor necrosis factor-α
